# 7-Chloro-5-(chloro­meth­yl)pyrazolo­[1,5-*a*]pyrimidine-3-carbonitrile

**DOI:** 10.1107/S160053681201166X

**Published:** 2012-03-24

**Authors:** Jingli Xu, Hang Liu, Guixia Li, Chuanmin Qi

**Affiliations:** a Key Laboratory of Radiopharmaceuticals, Ministry of Education, Department of Chemistry, Beijing Normal University, Xin Jie Kou Wai Street 19, 100875 Beijing, People’s Republic of China

## Abstract

All non-H atoms of the title compound, C_8_H_4_Cl_2_N_4_, are essentially coplanar, with an r.m.s. deviation of 0.011 Å. In the crystal, weak C—H⋯N hydrogen bonds link the mol­ecules into infinite sheets parallel to the *bc* plane.

## Related literature

For details of the synthesis, see: Li *et al.* (2006 ▶). For applications of pyrazolo­[1,5-*a*]pyrimidines as pharmacophores or building blocks in anti-tumor drug design, see: Li *et al.* (2006 ▶); Di Grandi *et al.* (2009 ▶); Powell *et al.* (2007 ▶); Gopalsamy *et al.* (2005 ▶).
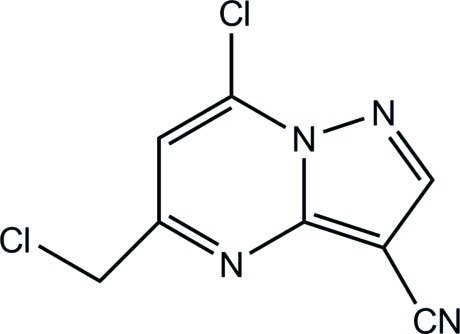


## Experimental

### 

#### Crystal data

C_8_H_4_Cl_2_N_4_
*M* *_r_* = 227.05
Monoclinic, 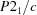

*a* = 4.9817 (4) Å
*b* = 18.4025 (15) Å
*c* = 10.1526 (9) Å
β = 95.924 (1)°
*V* = 925.78 (13) Å^3^
*Z* = 4
Mo *K*α radiation
μ = 0.66 mm^−1^
*T* = 301 K
0.60 × 0.48 × 0.20 mm

#### Data collection

Bruker SMART APEX CCD area-detector diffractometer
Absorption correction: multi-scan (*SADABS*; Bruker, 2007 ▶) *T*_min_ = 0.693, *T*_max_ = 0.879
5429 measured reflections
2111 independent reflections
1749 reflections with *I* > 2σ(*I*)
*R*_int_ = 0.017

#### Refinement

*R*[*F* ^2^ > 2σ(*F* ^2^)] = 0.038
*wR*(*F* ^2^) = 0.104
*S* = 1.04
2111 reflections
127 parameters
H-atom parameters constrained
Δρ_max_ = 0.46 e Å^−3^
Δρ_min_ = −0.53 e Å^−3^

### 

Data collection: *SMART* (Bruker, 1998 ▶); cell refinement: *SAINT* (Bruker, 1998 ▶); data reduction: *SAINT*; program(s) used to solve structure: *SHELXS97* (Sheldrick, 2008 ▶); program(s) used to refine structure: *SHELXL97* (Sheldrick, 2008 ▶); molecular graphics: *SHELXTL* (Sheldrick, 2008 ▶); software used to prepare material for publication: *SHELXTL*.

## Supplementary Material

Crystal structure: contains datablock(s) I, global. DOI: 10.1107/S160053681201166X/im2361sup1.cif

Structure factors: contains datablock(s) I. DOI: 10.1107/S160053681201166X/im2361Isup2.hkl

Supplementary material file. DOI: 10.1107/S160053681201166X/im2361Isup3.cml

Additional supplementary materials:  crystallographic information; 3D view; checkCIF report

## Figures and Tables

**Table 1:** Hydrogen-bond geometry (Å, °)

*D*—H⋯*A*	*D*—H	H⋯*A*	*D*⋯*A*	*D*—H⋯*A*
C8—H8⋯N2^i^	0.93	2.50	3.337 (3)	150
C2—H2⋯N2^ii^	0.93	2.70	3.515 (3)	146

Symmetry codes: (i) 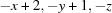; (ii) 
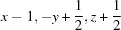
.

**Fig. 1.**
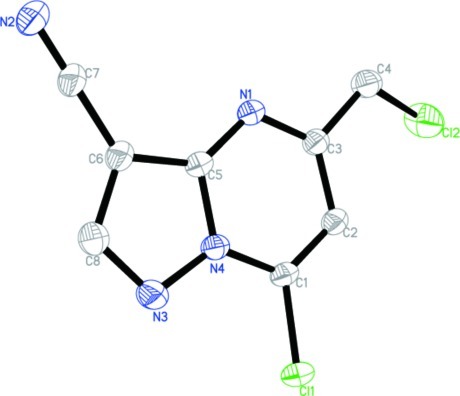
Molecular structure of the title compound showing displacement ellipsoids at the 45% probability level.

**Fig. 2.**
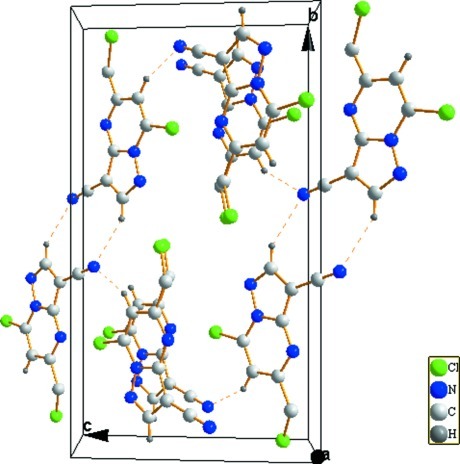
Packing diagram of the title compound, viewed along the a axis. Dashed lines indicate hydrogen bonds.

## supplementary crystallographic information

### Comment

Pyrazolo[1,5-*a*]pyrimidines are widely applied as important pharmacophores or building blocks in anti-tumor drug design (Di Grandi *et al.*, 2009; Powell *et al.*, 2007; Gopalsamy *et al.*, 2005; Li *et al.*, 2006). Thus, the synthesis of the title compound may lead to the development of further pyrazolo[1,5-*a*]pyrimidine derivatives as new anti-tumor drugs. Here we report the crystal structure of the title compound.

The molecular structure of the title compound is shown in Fig. 1. The complete molecule is essentially planar, except the H atoms of the methylene group. Each molecule acts as a donor and a acceptor of weak intermolecular C—H···N hydrogen-bond interactions linking the molecules into infinite sheets (Fig. 2).

### Experimental

The title compound can prepared by the reaction of 5-(chloromethyl)-7-hydroxypyrazolo[1,5-*a*]pyrimidine-3-carbonitrile with phosphorus oxychloride (Li *et al.*, 2006). Crystals suitable for X-ray diffraction were obtained by slow evaporation of a solution of the crude product in ethyl acetate at ambient temperature.

### Refinement

All H atoms attached to C atoms were fixed geometrically and treated as riding with C—H = 0.93 Å (CH) and C—H = 0.97 Å (CH_2_) with *U*_iso_(H) =1.2*U*_eq_(C).

### Crystal data

**Table:**

C_8_H_4_Cl_2_N_4_	*F*(000) = 456
*M**_r_* = 227.05	*D*_x_ = 1.629 Mg m^−^^3^
Monoclinic, *P*2_1_/*c*	Mo *K*α radiation, λ = 0.71073 Å
Hall symbol: -P 2ybc	Cell parameters from 2237 reflections
*a* = 4.9817 (4) Å	θ = 3.9–27.6°
*b* = 18.4025 (15) Å	µ = 0.66 mm^−^^1^
*c* = 10.1526 (9) Å	*T* = 301 K
β = 95.924 (1)°	Block, red
*V* = 925.78 (13) Å^3^	0.60 × 0.48 × 0.20 mm
*Z* = 4	

### Data collection

**Table:**

Bruker SMART APEX CCD area-detector diffractometer	2111 independent reflections
Radiation source: fine-focus sealed tube	1749 reflections with *I* > 2σ(*I*)
Graphite monochromator	*R*_int_ = 0.017
phi and ω scans	θ_max_ = 27.6°, θ_min_ = 2.3°
Absorption correction: multi-scan (*SADABS*; Bruker, 2007)	*h* = −6→6
*T*_min_ = 0.693, *T*_max_ = 0.879	*k* = −13→23
5429 measured reflections	*l* = −12→12

### Refinement

**Table:**

Refinement on *F*^2^	Primary atom site location: structure-invariant direct methods
Least-squares matrix: full	Secondary atom site location: difference Fourier map
*R*[*F*^2^ > 2σ(*F*^2^)] = 0.038	Hydrogen site location: inferred from neighbouring sites
*wR*(*F*^2^) = 0.104	H-atom parameters constrained
*S* = 1.04	*w* = 1/[σ^2^(*F*_o_^2^) + (0.050*P*)^2^ + 0.3703*P*] where *P* = (*F*_o_^2^ + 2*F*_c_^2^)/3
2111 reflections	(Δ/σ)_max_ < 0.001
127 parameters	Δρ_max_ = 0.46 e Å^−^^3^
0 restraints	Δρ_min_ = −0.53 e Å^−^^3^

### Special details

**Table:**

Geometry. All e.s.d.'s (except the e.s.d. in the dihedral angle between two l.s. planes) are estimated using the full covariance matrix. The cell e.s.d.'s are taken into account individually in the estimation of e.s.d.'s in distances, angles and torsion angles; correlations between e.s.d.'s in cell parameters are only used when they are defined by crystal symmetry. An approximate (isotropic) treatment of cell e.s.d.'s is used for estimating e.s.d.'s involving l.s. planes.
Refinement. Refinement of *F*^2^ against ALL reflections. The weighted *R*-factor *wR* and goodness of fit *S* are based on *F*^2^, conventional *R*-factors *R* are based on *F*, with *F* set to zero for negative *F*^2^. The threshold expression of *F*^2^ > σ(*F*^2^) is used only for calculating *R*-factors(gt) *etc*. and is not relevant to the choice of reflections for refinement. *R*-factors based on *F*^2^ are statistically about twice as large as those based on *F*, and *R*- factors based on ALL data will be even larger.

### Fractional atomic coordinates and isotropic or equivalent isotropic displacement parameters (Å^2^)

**Table:**

	*x*	*y*	*z*	*U*_iso_*/*U*_eq_	
Cl1	0.32935 (9)	0.26419 (3)	0.40816 (5)	0.04533 (17)	
Cl2	0.73176 (14)	0.03358 (3)	0.13931 (8)	0.0784 (3)	
N1	0.9223 (3)	0.23861 (8)	0.10690 (15)	0.0376 (3)	
N2	1.2065 (4)	0.41634 (10)	−0.0585 (2)	0.0636 (5)	
N3	0.5932 (4)	0.38077 (9)	0.26671 (18)	0.0503 (4)	
N4	0.6551 (3)	0.31043 (8)	0.24035 (15)	0.0374 (3)	
C1	0.5553 (4)	0.25052 (10)	0.29599 (18)	0.0363 (4)	
C2	0.6391 (4)	0.18420 (10)	0.25839 (18)	0.0390 (4)	
H2	0.5762	0.1420	0.2952	0.047*	
C3	0.8249 (4)	0.18073 (10)	0.16175 (18)	0.0378 (4)	
C4	0.9331 (5)	0.10975 (11)	0.1153 (2)	0.0554 (6)	
H4A	1.1109	0.1018	0.1615	0.066*	
H4B	0.9540	0.1138	0.0216	0.066*	
C5	0.8380 (3)	0.30370 (10)	0.14617 (17)	0.0352 (4)	
C6	0.8930 (4)	0.37484 (10)	0.11144 (19)	0.0423 (4)	
C7	1.0677 (4)	0.39787 (10)	0.0175 (2)	0.0473 (5)	
C8	0.7377 (5)	0.41835 (11)	0.1879 (2)	0.0520 (5)	
H8	0.7362	0.4688	0.1837	0.062*	

### Atomic displacement parameters (Å^2^)

**Table:**

	*U*^11^	*U*^22^	*U*^33^	*U*^12^	*U*^13^	*U*^23^
Cl1	0.0409 (3)	0.0559 (3)	0.0418 (3)	−0.0012 (2)	0.01678 (19)	−0.0018 (2)
Cl2	0.0764 (4)	0.0456 (3)	0.1169 (6)	−0.0135 (3)	0.0270 (4)	−0.0184 (3)
N1	0.0376 (8)	0.0389 (8)	0.0378 (8)	0.0000 (6)	0.0108 (6)	0.0019 (6)
N2	0.0747 (13)	0.0502 (11)	0.0710 (13)	−0.0039 (10)	0.0321 (11)	0.0098 (10)
N3	0.0591 (11)	0.0374 (9)	0.0574 (10)	0.0019 (8)	0.0213 (8)	−0.0043 (8)
N4	0.0381 (8)	0.0379 (8)	0.0377 (8)	−0.0003 (6)	0.0110 (6)	−0.0009 (6)
C1	0.0323 (8)	0.0449 (10)	0.0328 (8)	−0.0023 (7)	0.0089 (7)	0.0012 (7)
C2	0.0403 (9)	0.0381 (10)	0.0398 (9)	−0.0032 (8)	0.0099 (8)	0.0035 (8)
C3	0.0403 (9)	0.0364 (9)	0.0376 (9)	−0.0009 (7)	0.0075 (7)	0.0007 (7)
C4	0.0654 (14)	0.0379 (11)	0.0676 (14)	0.0002 (10)	0.0292 (11)	−0.0003 (10)
C5	0.0333 (8)	0.0391 (9)	0.0341 (9)	−0.0023 (7)	0.0078 (7)	0.0007 (7)
C6	0.0458 (10)	0.0368 (10)	0.0458 (10)	−0.0045 (8)	0.0114 (8)	0.0033 (8)
C7	0.0549 (12)	0.0360 (10)	0.0527 (12)	−0.0045 (9)	0.0137 (10)	0.0047 (9)
C8	0.0621 (13)	0.0350 (10)	0.0612 (13)	−0.0022 (9)	0.0177 (11)	−0.0011 (9)

### Geometric parameters (Å, º)

**Table:**

Cl1—C1	1.7006 (18)	C2—C3	1.418 (2)
Cl2—C4	1.755 (2)	C2—H2	0.9300
N1—C3	1.318 (2)	C3—C4	1.507 (3)
N1—C5	1.343 (2)	C4—H4A	0.9700
N2—C7	1.139 (3)	C4—H4B	0.9700
N3—C8	1.325 (3)	C5—C6	1.390 (3)
N3—N4	1.364 (2)	C6—C8	1.402 (3)
N4—C1	1.356 (2)	C6—C7	1.421 (3)
N4—C5	1.393 (2)	C8—H8	0.9300
C1—C2	1.357 (3)		
			
C3—N1—C5	117.09 (15)	C3—C4—H4A	108.6
C8—N3—N4	103.25 (16)	Cl2—C4—H4A	108.6
C1—N4—N3	126.15 (15)	C3—C4—H4B	108.6
C1—N4—C5	120.50 (15)	Cl2—C4—H4B	108.6
N3—N4—C5	113.35 (15)	H4A—C4—H4B	107.5
N4—C1—C2	118.49 (16)	N1—C5—C6	133.52 (16)
N4—C1—Cl1	117.07 (14)	N1—C5—N4	121.99 (15)
C2—C1—Cl1	124.44 (14)	C6—C5—N4	104.50 (15)
C1—C2—C3	118.48 (16)	C5—C6—C8	105.24 (17)
C1—C2—H2	120.8	C5—C6—C7	126.97 (18)
C3—C2—H2	120.8	C8—C6—C7	127.79 (19)
N1—C3—C2	123.45 (17)	N2—C7—C6	179.6 (3)
N1—C3—C4	114.14 (16)	N3—C8—C6	113.67 (19)
C2—C3—C4	122.40 (16)	N3—C8—H8	123.2
C3—C4—Cl2	114.86 (15)	C6—C8—H8	123.2
			
C8—N3—N4—C1	−179.43 (19)	C3—N1—C5—C6	−179.6 (2)
C8—N3—N4—C5	0.3 (2)	C3—N1—C5—N4	−0.2 (3)
N3—N4—C1—C2	−179.90 (18)	C1—N4—C5—N1	0.0 (3)
C5—N4—C1—C2	0.4 (3)	N3—N4—C5—N1	−179.71 (17)
N3—N4—C1—Cl1	0.6 (3)	C1—N4—C5—C6	179.58 (16)
C5—N4—C1—Cl1	−179.05 (13)	N3—N4—C5—C6	−0.1 (2)
N4—C1—C2—C3	−0.6 (3)	N1—C5—C6—C8	179.4 (2)
Cl1—C1—C2—C3	178.78 (14)	N4—C5—C6—C8	0.0 (2)
C5—N1—C3—C2	−0.1 (3)	N1—C5—C6—C7	0.0 (4)
C5—N1—C3—C4	−178.66 (18)	N4—C5—C6—C7	−179.5 (2)
C1—C2—C3—N1	0.5 (3)	N4—N3—C8—C6	−0.3 (3)
C1—C2—C3—C4	178.98 (19)	C5—C6—C8—N3	0.2 (3)
N1—C3—C4—Cl2	−159.03 (16)	C7—C6—C8—N3	179.7 (2)
C2—C3—C4—Cl2	22.4 (3)		

### Hydrogen-bond geometry (Å, º)

**Table:**

*D*—H···*A*	*D*—H	H···*A*	*D*···*A*	*D*—H···*A*
C8—H8···N2^i^	0.93	2.50	3.337 (3)	150
C2—H2···N2^ii^	0.93	2.70	3.515 (3)	146

Symmetry codes: (i) −*x*+2, −*y*+1, −*z*; (ii) *x*−1, −*y*+1/2, *z*+1/2.
